# Involvement of exercise-induced macrophage migration inhibitory factor in the prevention of fatty liver disease

**DOI:** 10.1530/JOE-13-0135

**Published:** 2013-08

**Authors:** Hyo Youl Moon, Parkyong Song, Cheol Soo Choi, Sung Ho Ryu, Pann-Ghill Suh

**Affiliations:** 1 BioSignal Network LaboratorySchool of Nano-Biotechnology and Chemical Engineering, Ulsan National Institute of Science and TechnologyEngineering Building 104689-805, UlsanRepublic of Korea; 1 Division of Molecular and Life SciencesPohang University of Science and Technology (POSTECH)Pohang, KyungbukRepublic of Korea; 2 Lee Gil Ya Cancer and Diabetes Institute and Gil Medical Center, Korea Mouse Metabolic Phenotyping CenterGachon UniversityIncheon, 406-840Republic of Korea

**Keywords:** cytokines, lipid, molecular biology, liver, exercise

## Abstract

Physical inactivity can lead to obesity and fat accumulation in various tissues. Critical complications of obesity include type II diabetes and nonalcoholic fatty liver disease (NAFLD). Exercise has been reported to have ameliorating effects on obesity and NAFLD. However, the underlying mechanism is not fully understood. We showed that liver expression of macrophage migration inhibitory factor (MIF) was increased after 4 weeks of treadmill exercise. Phosphorylation of AMP-activated protein kinase and acetyl-CoA carboxylase in human hepatocyte cell lines was enhanced after MIF treatment. These responses were accompanied by increases in lipid oxidation. Moreover, inhibition of either AMPK or cluster of differentiation 74 resulted in inhibition of MIF-induced lipid oxidation. Furthermore, the administration of MIF to a human hepatocyte cell line and mice liver reduced liver X receptor agonist-induced lipid accumulation. Taken together, these results indicate that MIF is highly expressed in the liver during physical exercise and may prevent hepatic steatosis by activating the AMPK pathway.

## Introduction

Excessive energy intake, including ingestion of a high-fat diet (HFD), and sedentary lifestyles cause hyperlipidemia, metabolic syndrome, and type II diabetes and are threatening to become a global epidemic (Angulo 2007, Lazo & Clark 2008). Nonalcoholic fatty liver disease (NAFLD) is the most common hepatic component of metabolic syndrome, which is highly associated with obesity and insulin resistance (Postic & Girard 2008). A representative phenomenon of NAFLD is hepatic accumulation of neutral lipids, mainly triglycerides (TGs), without ethanol consumption, viral infection, or other etiologies. NAFLD ranged from simple steatosis to cirrhosis accompanied by oxidative stress and hepatic injury (Angulo 2002). Although the precise mechanism of hepatic lipid accumulation remains incompletely understood, abnormal regulation of lipid disposal through fatty acid (FA) oxidation and processes affecting lipid availability, such as circulating free FA (FFA) uptake, have been suggested to be critical features (Browning & Horton 2004). Indeed, in HFD-induced hepatic steatosis, both elevated hepatic lipogenesis and impaired lipid oxidation contribute to hepatic TG accumulation (Postic & Girard 2008).

Hepatic lipid homeostasis is regulated by lifestyle modifications that aim to increase physical activity, induce weight reduction, and decrease energy intake (Farrell & Larter 2006). Exercise is accompanied by metabolic adaptations that promote the delivery to, and oxidation of, FAs in metabolic tissues. The mechanism by which exercise prevents hepatic steatosis involves AMP-activated protein kinase (AMPK; Lavoie & Gauthier 2006). AMPK is a conserved sensor of cellular energy and is activated under conditions in which energy is required, such as starvation and exercise, and by many endogenous hormones (Hardie 2007). Importantly, AMPK plays a critical role in lipid metabolism by regulating key substrates, including acetyl-CoA carboxylase (ACC) and FA synthase (FAS) (Ruderman & Prentki 2004). Based on the requirement for AMPK to alleviate hepatic steatosis, there is growing demand to identify mediators of AMPK activation in response to dynamic physiological activity.

Although macrophage migration inhibitory factor (MIF) has been identified as a pro-inflammatory cytokine (Nishio *et al*. 1999, Fingerle-Rowson & Bucala 2001), recent studies suggest that it has metabolic functions, including a protection of the heart during ischemia–reperfusion injury through effects on AMPK (Miller *et al*. 2008). In a previous study, we found that MIF expression was significantly increased in the hippocampus by long-term voluntary exercise (Moon *et al*. 2012). Based on the relationship between exercise and hepatic steatosis, we hypothesized that MIF may have a crucial role in the prevention of fatty liver disease during exercise. The purpose of this study was to determine the expression level of MIF after exercise in multiple metabolic tissues and to investigate whether MIF protects against hepatic steatosis. The potential effects of MIF on the AMPK activation and gene expression related to hepatic lipid metabolism were also investigated.

## Materials and methods

### Animals and treadmill exercise

For treadmill exercise experiments, male C57/BL6 (B6) mice (7–8 weeks old) were used. Mice were habituated under our laboratory condition for 2–3 days before initiation of the experiments. Totally, 19 mice were randomly assigned to two different groups. Mice in the exercise group (*n*=9) were acclimated to moderate treadmill running (10 m/min for 15 min) daily for 1 week. They were then subjected to 4 weeks of exercise training (5 days/week). The mice were trained on a treadmill inclined at 5°, with progressive increases in intensity and duration. At the end of 4 weeks, all exercise-trained mice were running for 50 min/day at a speed of 18 m/min. Electrical shock grids were used to stimulate the mice to run during treadmill running. The animal-handling procedures were based on the National Institutes of Health guidelines for animal studies. The experimental procedure was approved by the animal ethical review board of Lee Gil Ya Cancer and Diabetes Institute and Pohang Institute of Science and Technology. Each animal experiment used the minimum number of animals possible. The amount of MIF in serum was measured using a MIF ELISA kit (USCN Life Science, Inc., Wuhan, China). The blood was collected from orbital sinus in the mice using previously described protocol. According to previous papers (Santos *et al*. 2001, Gao & Liu 2013, Vitzel *et al*. 2013), treatment of mice with vehicle (saline) or T0901317 (T090; Sigma) 5 mg/kg was performed by daily i.p. injection for 5 consecutive days. AICAR (0.4 g/kg, TRC, Toronto, ON, Canada) or rMIF (1 mg/kg;) with T0901317 (T090; Sigma) 5 mg/kg was administrated by daily single i.p. injection for 5 consecutive days.

### Cell culture

HepG2 cells were maintained in MEM (Sigma Chemical Company) containing 100 mg/ml kanamycin supplemented with 10% fetal bovine serum (FBS; Sigma Chemical Company) at 37 °C in 100 mm cell culture dishes (Corning, Inc., Oneonta, NY, USA) under a humidified atmosphere containing 5% CO_2_ in air. *CD74* siRNA (catalog no. sc-35024) and control scramble siRNA (catalog no. sc-37007) were purchased from Santa Cruz Biotechnology. A total of 2×10^4^ cells/well for HepG2 and 2×10^6^ cells/well for HepG2 were plated for 24 h, and siRNA transfection was conducted using Lipofectamine 2000 according to the manufacturer's instructions (Invitrogen).

### Primary hepatocyte culture

Primary hepatocytes were isolated by collagenase digestion, following a previously described protocol with small modification (Klaunig *et al*. 1981, Yanhong *et al*. 2008). Briefly, mice were anesthetized and the portal vein was cannulated with a 22-gauge i.v. catheter. The liver was perfused with Hank's buffer solution (Invitrogen) containing 0.5 mM EGTA and 0.05 M HEPES (pH 7.4) maintained at 37 °C at a rate of 5 ml/min for 5 min. Then, the collagenase-containing solution (Hyclone Medium 199/EBSS (Thermo Scientific, Logan, UT, USA) with 0.05% collagenase Type IA (Sigma)) was used for the perfusion for 5 min (5 ml/min). The liver was transferred to 10 mm dishes with 15 ml DMEM containing 0.05% collagenase and was mechanically dissociated into single cells. Ten milliliters DMEM supplemented with 10% heat-inactivated FBS were added to the cells to reduce collagenase activity. Cells were filtered through a 70 μm pore size strainer (BD, San Jose, CA, USA) and centrifuged at 120 ***g*** (Percoll gradient centrifugation) for three times. The cell yield was counted using a hemocytometer and the viability of the cells was assessed using Trypan blue exclusion test.

### Western blot analysis

The HepG2 cells were grown in six-well plates. After reaching 60–70% confluence, the cells were fasted for 24 h prior to treatment with the selected agents and incubation at 37 °C. The medium was aspirated, the cells were washed twice in ice-cold PBS, and then lysed in 100 μl of lysis buffer (0.5% deoxycholate, 0.1% SDS, 1% Nonidet P-40, 150 mM NaCl, and 50 mM Tris–HCl (pH 8.0)) containing proteinase inhibitors (0.5 μM aprotinin, 1 μM phenylmethylsulfonyl fluoride, and 1 μM leupeptin) (Sigma Chemical Company). The supernatants were sonicated briefly, heated for 5 min at 95 °C, centrifuged for 5 min, separated on SDS–PAGE (8–16%) gels, and finally transferred to polyvinylidene difluoride membranes. The blots were then incubated overnight at 4 °C with primary antibodies and washed six times in Tris-buffered saline/0.1% Tween 20 prior to probing with HRP-conjugated secondary antibodies for 1 h at room temperature. Anti-phospho-AMPK, anti-MIF, anti-ACC, and anti-AMPK antibodies were purchased from Cell Signaling Technology (New England Biolabs, Beverly, MA, USA). Anti-phospho-ACC was purchased from Upstate (Waltham, MA, USA). To normalize protein loading, an anti-β-actin antibody (MP Biomedical, Solon, OH, USA) was used for blotting. The blots were then visualized with ECL (GE Biosciences, Piscataway, NJ, USA).

### Adenoviral transfection of a dominant-negative AMPK2 isoform

Recombinant adenoviral vectors expressing a myc-tagged dominant-negative mutant of *AMPK2* and a control virus were generated as described previously (Lee *et al*. 2003). HepG2 cells were infected with the indicated multiplicity of infection (MOI, or viral particle:cell ratio) titers for 6 h in serum-free MEM. The cells were then washed and incubated in growth medium for 12 h.

### Real-time RT-PCR

Total RNA was extracted using a total RNA extraction kit (iNtRON Biotechnology, Seoul, Korea). First-strand cDNA was synthesized by RT using oligo(dT) primers and SuperScript II reverse transcriptase (Invitrogen). cDNA was amplified for 25–30 cycles using mouse or rat gene-specific primers (Table 1). For real-time RT-PCR, total RNA (100 ng) was amplified using the One-Step SYBR RT-PCR kit and a Light Cycler 2.0 PCR system (Roche Diagnostics).

### Palmitate oxidation assay

After starvation for 2 h, HepG2 cells were incubated in oxidation medium containing 0.1 mmol/l palmitate (9,10-[^3^H] palmitate, 5 μCi/ml) and 0.1% lipid-free BSA. After oxidation, the medium was precipitated with the same volume of 10% TCA solution. The supernatants were transferred to capless tubes placed in a scintillation vial containing 0.5 ml of unlabeled water and were incubated at 50 °C for 12 h. After evaporation and equilibration, the tubes were removed and scintillation enhancer fluid (PerkinElmer Life and Analytical Sciences, Waltham, MA, USA) was added to the vial.

### Determination of hepatic lipid contents

Quantitative measurements of hepatic TG accumulation in HepG2 cells were carried by lipid extraction using chloroform and enzymatic assays using the EnzyChrom triglyceride assay kit (Bioassay Systems, Hayward, CA, USA).

### Statistical analysis

All data are expressed as mean±s.e.m. and are representative of at least three different experiments. Data were analyzed by two-tailed Student's *t*-test or one-way ANOVA followed by an LSD *post hoc* test. In all cases, *P* values of <0.05 were deemed to be statistically significant. Statistical analysis was performed using SPSS 17.0 (SPSS Corp.).

## Results

### Expression profiling of MIF after 4 weeks of treadmill exercise

**Figure 1:**
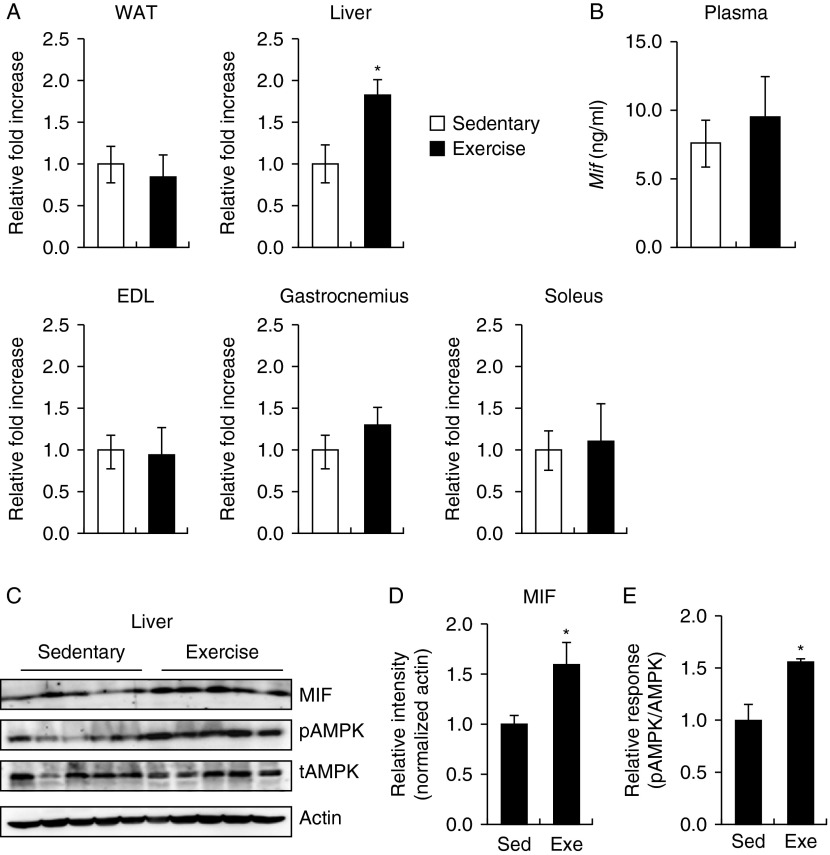
Expression of MIF in sedentary (Sed) and exercised (Exe) mice. (A) RT-PCR analysis of MIF mRNA in various metabolic tissues. *18s* rRNA levels were used as a control. (B) ELISA analysis of plasma MIF levels in mice. (C) Lysates from exercised or sedentary mouse tissues were subjected to western blotting using anti-phospho-AMPK, anti-total AMPK, and anti-MIF antibodies. Anti-β-actin antibodies were used to confirm equal protein loading. (D) Bar graph depicts the mean (±s.e.m.) ratio of intensity of MIF-to-actin bands and phospho-AMPK-to-total AMPK bands. (E) The levels of phosphorylated AMPK in liver were measured after exercise. Data are presented as the mean±s.e.m. (Figures are representative of ten sedentary and nine exercised mouse samples). *P<0.05 vs control values

To determine whether MIF is involved in metabolic effects during exercise, we evaluated the expression level of MIF in various metabolic tissues and plasma using a mouse treadmill running model. We confirmed by real-time PCR that liver MIF expression was significantly increased after 4 weeks of treadmill running; MIF expression in white adipose tissue, the soleus, the extensor digitorum longus, and the gastrocnemius was unchanged (Fig. 1A). We detected a marginal increase in plasma MIF level after exercise by ELISA (Fig. 1B). Even though the expression level of cluster of differentiation 74 (CD74), one of the MIF's receptors, was not significantly changed (Supplementary Figure 1A, see section on supplementary data given at the end of this article), we also observed that the MIF level was upregulated in the exercise group when compared with the sedentary group (Fig. 1C and D). Finally, western blotting analysis showed that phosphorylation of AMPK was increased in the exercise group compared with sedentary group in liver (Fig. 1E), as described in the previous study (Takekoshi *et al*. 2006).

### MIF stimulates the AMPK pathway in hepatocytes

**Figure 2:**
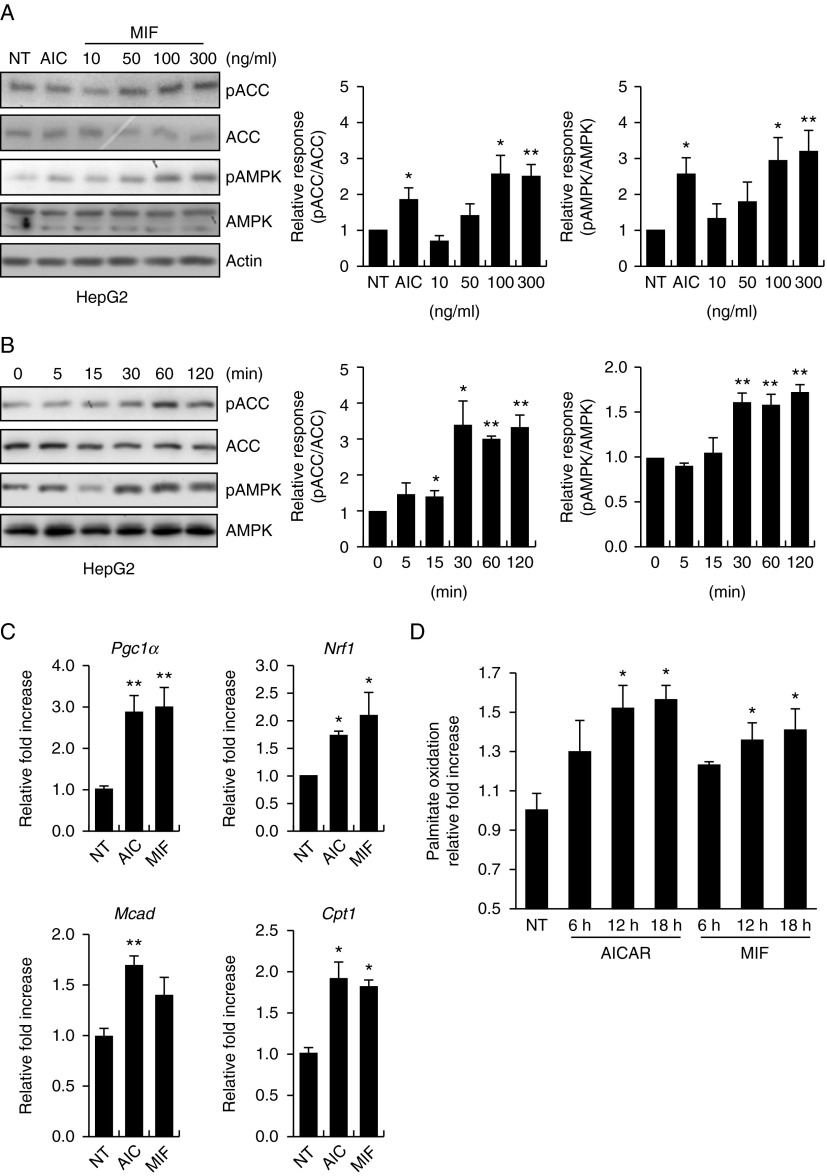
MIF activates AMPK–ACC, stimulates palmitate oxidation, and increases mitochondria-related gene expression in HepG2 cells. (A) Dose-dependent phosphorylation of AMPK by MIF. HepG2 cells were stimulated with the indicated doses of MIF, AICAR, or vehicle for 1 h. Cell lysates were analyzed by western blotting with anti-phospho-ACC (Ser79) and anti-phospho-AMPK (Thr72) antibodies. Anti-ACC, anti-AMPK, and anti-β-actin antibodies were to check protein loadings. (B) Time-dependent phosphorylation of AMPK by MIF. HepG2 cells were stimulated with MIF (100 ng/μl) for the indicated periods of time. Bar graph depicts the mean (±s.e.m.) ratio of intensity of phospho-ACC-to-total ACC bands and phospho-AMPK-to-total AMPK bands. (C) HepG2 cells were incubated in six-well plates for 24 h with vehicle, MIF (100 ng/μl), or AICAR (100 nM). After 24 h, whole-cell lysates were isolated for analysis of mRNA expression of *Pgc1α*, *Nrf1*, *Mcad*, and *Cpt1*. (D) HepG2 cells were incubated in 60 mm dishes for 24 h with vehicle, MIF, and AICAR. After washing, cells were assayed for oxidation of [^3^H]-labeled palmitate, as described in the Materials and methods section. *P<0.05 vs control values (one-way ANOVA). **P<0.01 vs control values. Data are expressed as mean±s.d. of triplicate analyses.

The administration of MIF induced a dose- and time-dependent increase in AMPK phosphorylation in HepG2 cells (Fig. 2A and B). AMPK phosphorylation reached a maximum level after treatment with 100 ng/μl MIF for 60 min. Consistent with the increase in AMPK phosphorylation, the phosphorylation of ACC, a downstream target of AMPK, also increased after MIF administration. AICAR (1 mM), a known AMPK activator, was used as a positive control (Table 1).

### Effect of MIF on lipid oxidation

AMPK accomplishes the transition to the oxidative mode of metabolism by upregulating genes related to mitochondrial biosynthesis and oxidative enzymes such as nuclear respiratory factor 1 (NRF1), medium-chain acyl-CoA dehydrogenase (MCAD), carnitine palmitoyltransferase-1 (CTP1b), and peroxisome proliferator-activated receptor-γ coactivator-1α (PGC1α) (Tian *et al*. 2001, Lee *et al*. 2006, Li *et al*. 2007). We next tested whether MIF treatment affected the expression of genes related to mitochondrial metabolism and FA utilization. MIF robustly induced the expression of *PGC1α, NRF*, and *CPT1* in HepG2 cells (Fig. 2C). To further characterize the mechanism underlying MIF-mediated AMPK activation, we performed palmitate oxidation analysis. We observed that MIF increased palmitate oxidation in HepG2 cells at a concentration of 100 ng/μl (Fig. 2D). AICAR (100 nM) also increased palmitate oxidation and was utilized as a positive control. These results suggest that MIF-mediated AMPK activation may be associated with lipid oxidation.

### Inhibition of AMPK suppresses MIF-induced lipid oxidation

**Figure 3:**
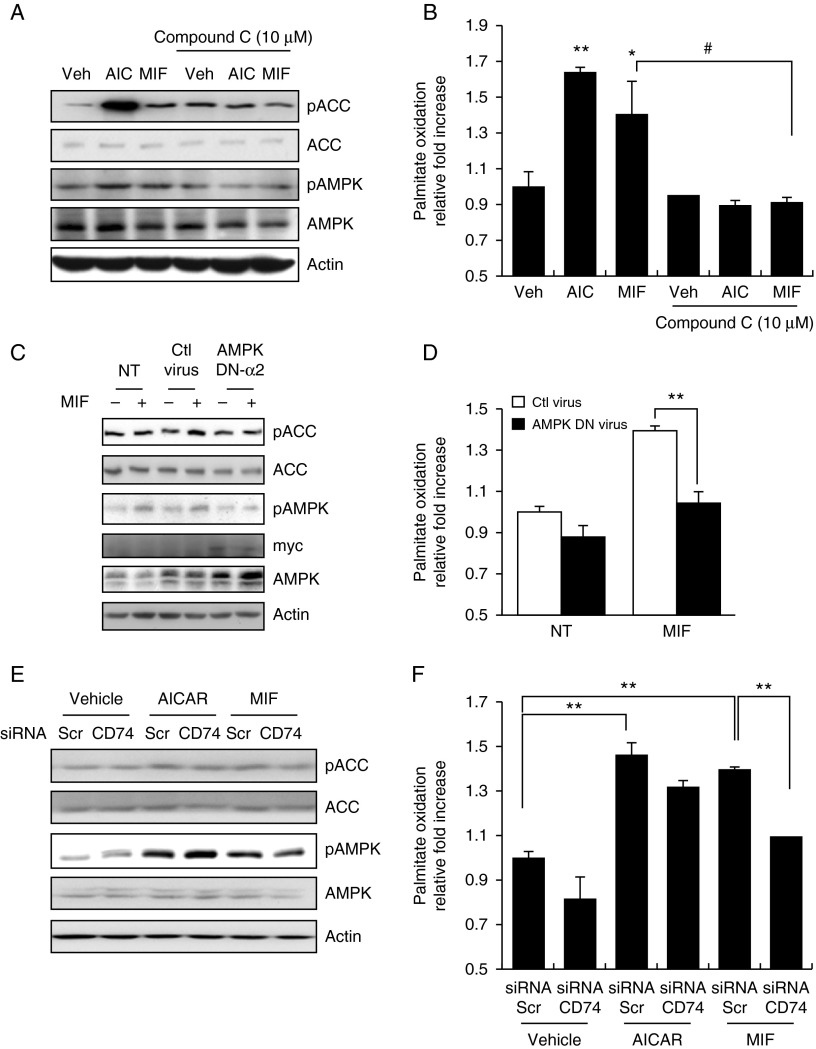
MIF-stimulated palmitate oxidation in a CD74–AMPK-dependent manner. (A) HepG2 cells were pre-treated with compound C (10 μM) for 30 min and were then stimulated with MIF or AICAR for 1 h. Cell lysates were analyzed by western blotting with an anti-phospho-AMPK(Thr72) antibody. (B) Cells were pre-incubated with compound C and then stimulated under the indicated conditions. Oxidation of [^3^H]-labeled palmitate was measured as described in the Materials and methods section. (C) Cells infected with a mock adenovirus or an adenovirus carrying dominant-negative (DN)-AMPK α2 at an MOI of 30 for 18 h were treated with or without MIF. Cell lysates were analyzed by western blotting. DN-AMPK α2 expression was confirmed using an anti-Myc antibody. (D) Oxidation of [^3^H]-labeled palmitate was measured after infection with the mock or DN-AMPK a2 adenovirus. (E) HepG2 cells were transfected with CD74 or scrambled siRNA for 48 h and were then stimulated with MIF for 24 h. Whole-cell lysates were used to detect the phosphorylation of AMPK and ACC. (F) HepG2 cells were transfected with CD74 or scrambled siRNA for 48 h and were then stimulated with MIF for 24 h. HepG2 cells were incubated in 60 mm dishes for 24 h with either MIF or AICAR and were then assayed for oxidation of [^3^H]-labeled palmitate. Data are expressed as means±s.d. of triplicate experiments. *P< 0.05 vs vehicle; **P< 0.01 vs vehicle values and #*P*< 0.05 vs NT (compound C not treated) values (one-way ANOVA).

To examine the dependency of MIF-induced FA oxidation on AMPK, we used a selective AMPK chemical inhibitor and virus carrying a dominant-negative form of AMPK α2, which was shown to be a major isoform in skeletal muscle. As shown in Fig. 3A and Supplementary Figure 2A, see section on supplementary data given at the end of this article, MIF-induced AMPK activation was completely blocked by pretreatment with the AMPK inhibitor compound C in HepG2 and primary mouse hepatocytes. Pretreatment with compound C (10 μM) also inhibited MIF-induced palmitate oxidation, indicating that the AMPK pathway is involved in MIF-induced lipid metabolism in HepG2 and primary mouse hepatocytes (Fig. 3B and Supplementary Figure 2B). To confirm that the effects of MIF were mediated by AMPK activation, we investigated the effects of MIF on palmitate oxidation by over-expressing either dominant-negative AMPK or wild-type AMPK in hepatocytes. Expression of AMPK viruses was confirmed by blotting with an antibody against Myc, which was used to tag the virus. MIF-induced AMPK phosphorylation and palmitate oxidation were clearly reduced in the cells infected with the dominant-negative form of AMPK (Fig. 3C, D and Supplementary Figure 3B, C). AMPK is thus important for MIF to induce lipid oxidation in the liver.

### A CD74–AMPK pathway is involved in MIF-induced lipid oxidation

To elucidate the role of CD74 in MIF-induced lipid metabolism, we assessed the effects of a kinase inhibitor and CD74-specific siRNAs on palmitate oxidation. MIF-induced phosphorylation of AMPK and ACC in HepG2 cells was attenuated by the knockdown of *Cd74* expression (Fig. 3E). Moreover, inhibition of *Cd74* expression by siRNAs (Supplementary Figure 3A) reduced MIF-induced palmitate oxidation in HepG2 cells (Fig. 3F). Viewed together, our findings appear to show that AMPK pathways play an important role in MIF-induced lipid oxidation.

### MIF inhibits lipid accumulation due to liver X receptor agonist exposure

**Figure 4:**
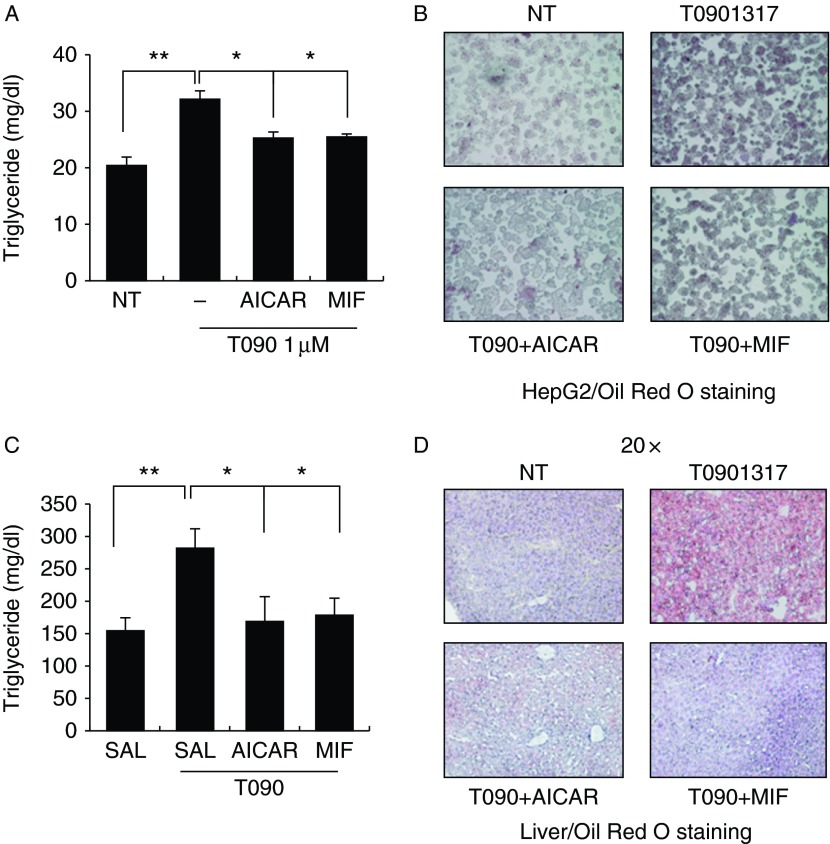
Hepatic lipid accumulation was inhibited by MIF treatment. (A) Intracellular TG contents were measured under the indicated conditions. HepG2 cells were exposed to T0901317, a synthetic LXRα ligand, with or without MIF for 24 h. Lipid levels were determined by the TG hydrolysis method. (B) Cells were treated with AICAR or MIF under conditions of T0901317 stimulation. After 24 h, the cells were stained with Oil Red O to observe the accumulation of lipids. Data are expressed as means±s.d. of triplicate experiments. (C) Mice were administrated with vehicle (saline), AICAR, or MIF under conditions of T0901317 (*n*=4) stimulation. Intracellular TG contents were measured after 5 days of administration. Lipid levels were determined by the TG hydrolysis method. *P<0.05 vs control values, **P<0.01 vs control values. (D) The mice liver of the each experimental group was prepared for cryosection with optimal cutting temperature compound and stained with Oil Red O and hematoxylin to observe the accumulation of lipids. Data are presented as mean±s.e.m.

To determine whether increased FA oxidation can be linked to intracellular TG levels, we measured total TG levels by lipid extraction methods after co-treatment with MIF plus an liver X receptor (LXR) agonist (1 μM). Similar to the lipid oxidation results, treatment for 24 h decreased cellular TG levels to levels comparable with those in AICAR controls (Fig. 4A). To further confirm the TG decreases, we stained HepG2 cells and primary mouse hepatocytes with Oil Red O after MIF treatment. LXR agonist-treated cells contained large neutral lipid stores. MIF treatment greatly attenuated the Oil Red O signal in HepG2 and primary mouse hepatocytes (Fig. 4B and Supplementary Figure 2C). Furthermore, *Fasn*, one of the LXR target genes, was reduced by MIF treatment, and pretreatment of compound C recovers the effect of MIF on *Fasn* gene expression in primary mouse hepatocytes (Supplementary Figure 4A, see section on supplementary data given at the end of this article). Consistent with these findings, administration of rMIF with LXR agonist substantially decreased the FA accumulation in the liver compared with LXR agonist-only treated group (Fig. 4C, D and Supplementary Figure 5A). These findings demonstrate that MIF can prevent LXR agonist-induced hepatic lipid accumulation.

## Discussion

Although exercise and AMPK are regarded as key factors in combating NAFLD, little is known about endogenous mediators contributing to the improvement. In the present report, the most striking and unexpected finding was the MIF level in the liver after 4 weeks of treadmill exercise. Under hypoxic or ischemic conditions, MIF may be released from several tissues, such as endothelial progenitor cells or the heart (Miller *et al*. 2008, Simons *et al*. 2011). Hypoxic conditions are readily induced by exercise (Moller *et al*. 2001), which may explain how exercise induced MIF expression. However, we observed that the circulating MIF level in the exercise group was not significantly different from that in the control group. Based on our data and a previous report (Miller *et al*. 2008), it seems that MIF reuptake into the liver or other organs may explain the unchanged serum MIF level. Further studies are needed to elucidate this mechanism.

Importantly, MIF activates AMPK, resulting in the amelioration of lipid accumulation of hepatocytes. We described a putative mechanism by which MIF exerts its steatosis-preventing effect by demonstrating AMPK-dependent lipid oxidation in HepG2 cells. Through intracellular TG measurement and Oil Red O staining, we demonstrated that MIF administration inhibited chemically induced lipid accumulation in mice liver and human hepatoblastoma cell lines. Together, these results demonstrate that MIF may mediate the preventive effect of exercise against hepatic steatosis through AMPK.

Both isoforms of ACC (ACC1 and ACC2) are independently involved in lipid metabolism (Brownsey *et al*. 2006). ACC1 takes charge of *de novo* lipogenesis, while ACC2 is thought to negatively regulate FA oxidation by modulating local malonyl-CoA levels (Wakil *et al*. 1983, McGarry & Brown 1997). Although MIF-induced FA oxidation is important for alleviating hepatic steatosis, MIF-induced inhibition of lipogenesis in the liver may be another reasonable mechanism for TG regulation. Sterol regulatory element-binding protein (SREBP) is a transcription factor that binds to the sterol regulatory element and regulates the expression of lipogenic enzymes, such as stearoyl-CoA desaturase-1, FAS, and ACC1 (Brown & Goldstein 1997, Postic & Girard 2008). Prolonged treatment with MIF plus an LXR agonist induced a significant decrease in the mRNA expression of SREBP-1C, which is mainly involved in hepatic lipogenesis (data not shown). Thus, MIF may have a dual function, suppressing lipid accumulation by modulating lipogenesis and lipid oxidation simultaneously.

HLA class II histocompatibility antigen gamma chain, also known as CD74, mediates MIF-triggered signaling (Leng *et al*. 2003, Lue *et al*. 2006, Heinrichs *et al*. 2011), which plays various roles in cell-mediated immunity, inflammation, cell growth, and some neuronal processes (Lewis *et al*. 1998). *Cd74* expression was recently examined in various cell types other than antigen-presenting cells, including liver cells, and is particularly important in complex immunological functions and in the link between chronic inflammation and liver disease (Koch & Leffert 2011). Surprisingly, expression of *Cd74* is closely related to inhibition of fibrogenesis, which can protect against liver cirrhosis (Heinrichs *et al*. 2011). We showed that MIF induced activation of AMPK, and that inhibition of CD74 reduced the effect of MIF on AMPK activation and palmitate oxidation in HepG2 cells. These results suggest that a CD74–AMPK pathway is involved in metabolic effects following MIF treatment.

In summary, we suggest that MIF stimulates lipid oxidation in the liver via a CD74–AMPK pathway. These findings increase our understanding of how exercise-induced factors ameliorate hyperlipidemia during exercise. Taken together, our findings identify an important mediator that may reduce lipid accumulation in response to exercise.

## Supplementary data

This is linked to the online version of the paper at http://dx.doi.org/10.1530/JOE-13-0135.

Supplemental Data

## Figures and Tables

**Table 1:** List of primer sequences used for RT-PCR analysis in this study.

**Gene**	**Sequence**	**Direction**	**Species**
*PGC1α*	GTAAATCTGCGGGATGATGG	Sense	Human
	ATTGCTTCCGTCCACAAAA	Antisense	
*CPT1b*	ATCCTTGCTTGTGGGAACAG	Sense	Human
	TCCATGCTGACAAGAAGCTG	Antisense	
*MCAD*	TGCCCTGGAAAGGAAAACTTT	Sense	Human
	GTTCAACTTTCATTGCCATTTCAG	Antisense	
*NRF-1*	CCACGTTACAGGGAGGTGAG	Sense	Human
	TGTAGCTCCCTGCTGCATCT	Antisense	
*18S rRNA*	TCGGCGTCCCCCAACTTCTTA	Sense	Mouse
	GGTAGTAGCGACGGGCGGTGT	Antisense	
*Mif*	ACAGCATCGGCAAGATCG	Sense	Mouse
	GGCCACACAGCAGCTTACT	Antisense	
*Fasn*	CATCCAGATAGGCCTCATAGAC	Sense	Mouse
	CTCCATGAAGTAGGAGTGGAAG	Antisense	
