# Supplementary material for: Involvement of exercise-induced macrophage migration inhibitory factor in the prevention of fatty liver disease
Source: J Endocrinol. 2013 Jul 3;218(3):339–48. doi: 10.1530/JOE-13-0135 (PMC3757527; doi:10.1530/JOE-13-0135)
Supplement: Supplemental Data [file supp_218_3_339__index.html]

Supplemental Data 

# Involvement of exercise-induced macrophage migration inhibitory factor in the prevention of fatty liver disease

## Supplementary Data

**Files in this Data Supplement:**

- Supplementary Figure 1 - Expression of CD74 in sedentary and exercised mice. (A) RT-PCR analysis of CD74 mRNA in the liver of sedentary and exercised mice. 18s rRNA levels were used as a control. Data are presented as the mean ± SE (Figures are representative of 10 sedentary and 9 exercised mouse samples). (PDF 12 KB)
- Supplementary Figure 2 - MIF activate AMPK-ACC through phosphorylation and stimulated palmitate oxidation in an AMPK-dependent manner in the primary hepatocytes. (A) Primary hepatocytes were pre-treated with compound C (10 μM) for 30 min and were then stimulated with MIF or AICAR for 1 h. Cell lysates were analyzed by Western blotting with an anti-phospho-AMPK(Thr72) antibody. (B) Cells were pre-incubated with compound C and then stimulated under the indicated conditions. Oxidation of [3H]-labeled palmitate was measured as described in the Materials and Methods. (C) Primary hepatocytes were treated with AICAR or MIF under conditions of TO901317 stimulation. After 24 h, the cells were stained with Oil Red O to observe the accumulation of lipids. (PDF 69 KB)
- Supplementary Figure 3 - MIF stimulated palmitate oxidation in a CD74-AMPK-dependent manner. (A) RTPCR analysis of cd74 mRNA in the HepG2 cells transfected with scramble (Scr) siRNA or CD74 siRNA. 18s rRNA levels were used as a control. (B) HepG2 cells were pre-treated with compound C (10 μM) for 30 min and were then stimulated with MIF or AICAR for 1 h. Cell lysates were analyzed by Western blotting with an antiphospho-AMPK(Thr72) antibody. (C) Cells infected with a mock adenovirus or an adenovirus carrying dominant-negative (DN)-AMPK α2 at an MOI of 30 for 18 h were treated with or without MIF. Cell lysates were analyzed by Western blotting. (B, C) Bar graph depicts the mean (+ SE) ratio of intensity of phospho AMPK- to-total AMPK bands. \*p < 0.05, \*\*p < 0.01 vs. control values (one-way ANOVA). Data are expressed as the means ± SD of quadruplicated analyses. (PDF 55 KB)
- Supplementary Figure 4 - Effect of MIF on the expression of Fasn. (A) HepG2 cells were pre-treated with compound C (10 μM) for 30 min and were then stimulated with MIF or AICAR for 1 h. Cell lysates were analyzed by RT-PCR analysis with Fasn specific primer. 18s rRNA levels were used as a control. \*\*p < 0.01 vs. control values (one-way ANOVA). Data are expressed as the means ± SD of triplicate analyses. (PDF 114 KB)
- Supplementary Figure 5 - (A) Mice were administrated with Vehicle (saline), AICAR or MIF under conditions of TO901317 (n=4, respectively) stimulation. Intracellular TG contents were measured after 5 day of administration. The mice liver of the each experimental group were prepared for cryosection with optimal cutting temperature (OCT) compound and stained with Oil Red O and hematoxlyin to observe the accumulation of lipids using microscopy (IX71, Olympus, Japan). (PDF 101 KB)
