# Supplementary material for: Involvement of exercise-induced macrophage migration inhibitory factor in the prevention of fatty liver disease
Source: J Endocrinol. 2013 Jul 3;218(3):339–48. doi: 10.1530/JOE-13-0135 (PMC3757527; doi:10.1530/JOE-13-0135)
Supplement: Supplemental Data [file supp_JOE-13-0135_Supplementary_figure_1.pdf]

Supplementary Fig 1.

A.

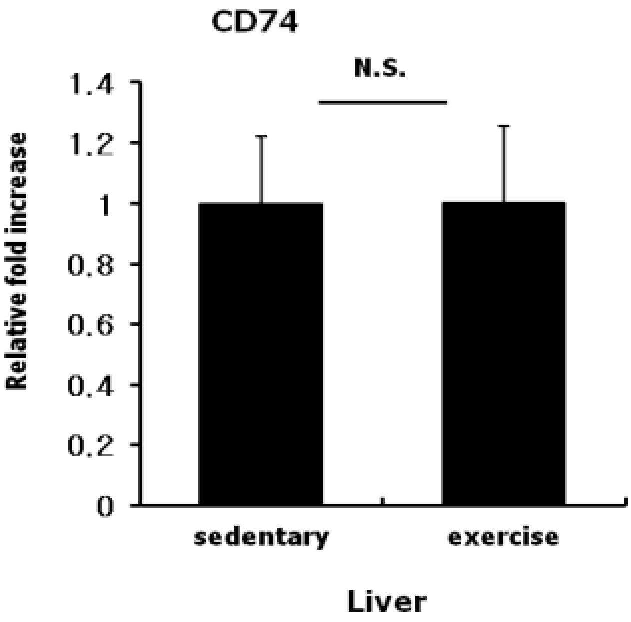

Supplementary Fig. 1. Expression of CD74 in sedentary and exercised mice. (A) RT-PCR analysis of CD74 mRNA in the liver of sedentary and exercised mice. 18s rRNA levels were used as a control. Data are presented as the mean ± SE (Figures are representative of 10 sedentary and 9 exercised mouse samples).
