# Supplementary material for: Involvement of exercise-induced macrophage migration inhibitory factor in the prevention of fatty liver disease
Source: J Endocrinol. 2013 Jul 3;218(3):339–48. doi: 10.1530/JOE-13-0135 (PMC3757527; doi:10.1530/JOE-13-0135)
Supplement: Supplemental Data [file supp_JOE-13-0135_Supplementary_figure_2.pdf]

Supplementary Fig 2.

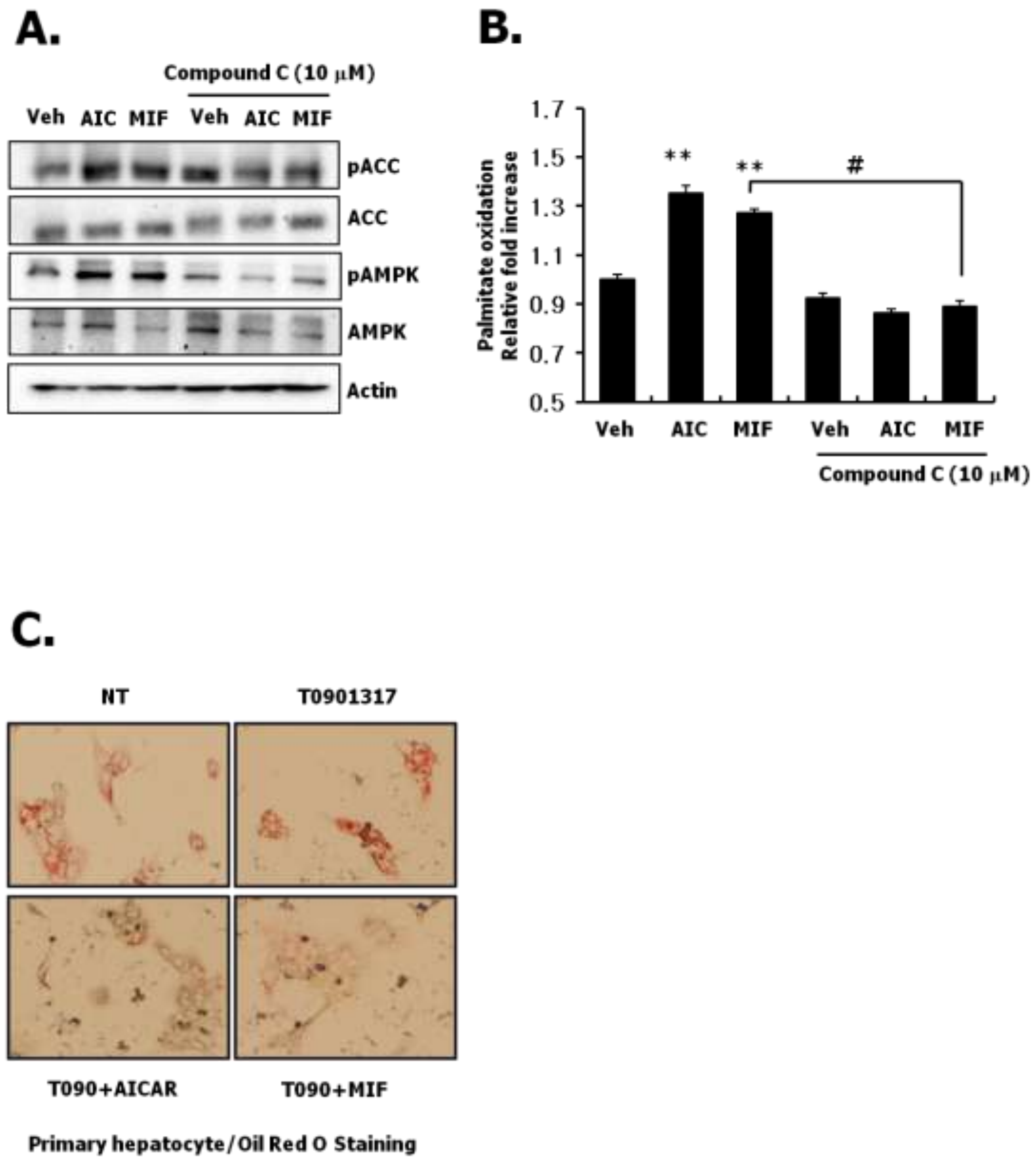

Supplementary Fig. 2. MIF activate AMPK-ACC through phosphorylation and stimulated palmitate oxidation in an AMPK-dependent manner in the primary hepatocytes. (A) Primary hepatocytes were pre-treated with compound C (10 μM) for 30 min and were then stimulated with MIF or AICAR for 1 h. Cell lysates were analyzed by Western blotting with an anti-phospho-AMPK(Thr72) antibody. (B) Cells were pre-incubated with compound C and then stimulated under the indicated conditions. Oxidation of [^{3}H]-labeled palmitate was measured as described in the Materials and Methods. (C) Primary hepatocytes were treated with AICAR or MIF under conditions of TO901317 stimulation. After 24 h, the cells were stained with Oil Red O to observe the accumulation of lipids.
