# Supplementary material for: Involvement of exercise-induced macrophage migration inhibitory factor in the prevention of fatty liver disease
Source: J Endocrinol. 2013 Jul 3;218(3):339–48. doi: 10.1530/JOE-13-0135 (PMC3757527; doi:10.1530/JOE-13-0135)
Supplement: Supplemental Data [file supp_JOE-13-0135_Supplementary_figure_3.pdf]

Supplementary Fig 3.

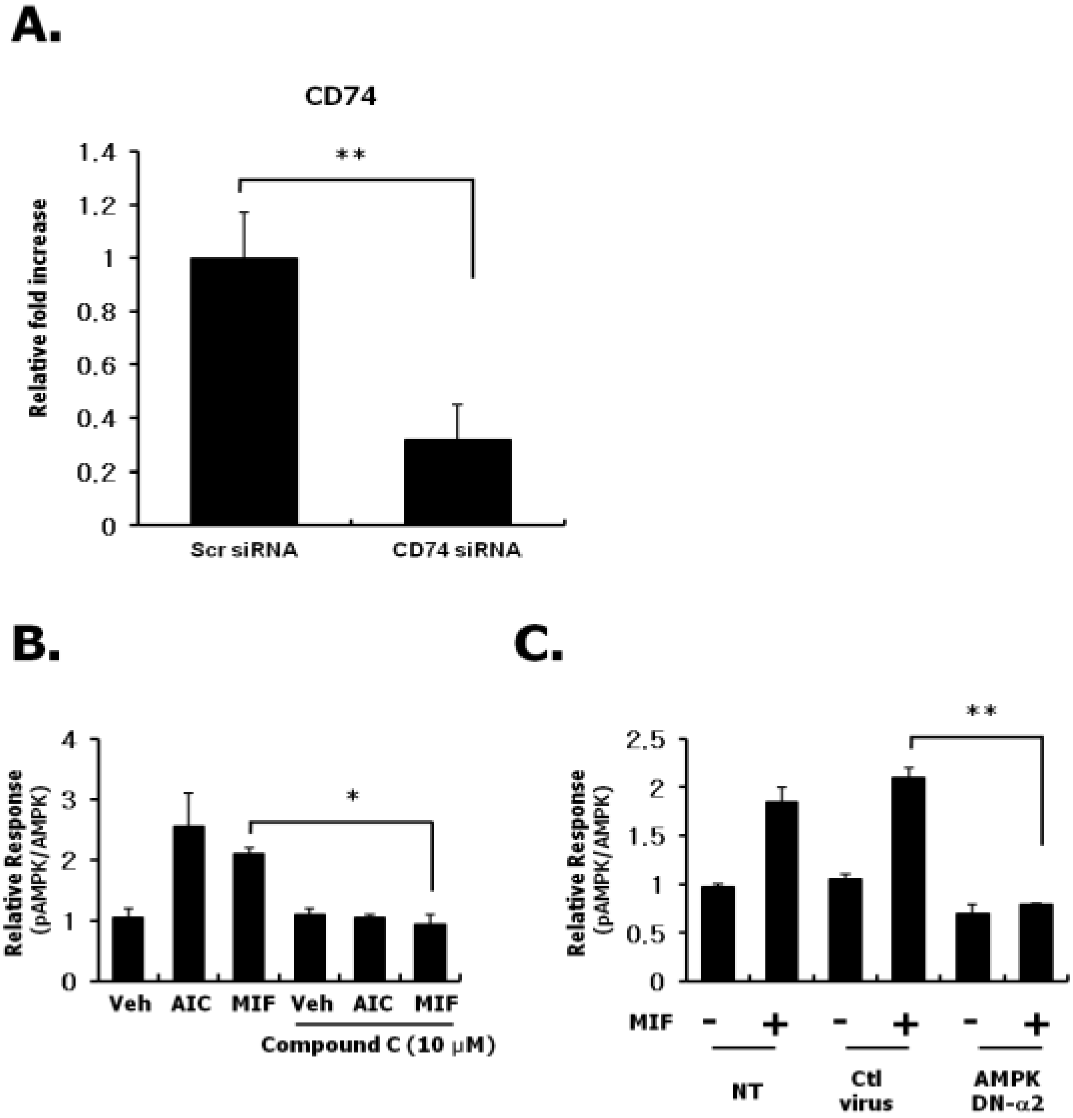

Supplementary Fig. 3. MIF stimulated palmitate oxidation in a CD74-AMPK-dependent manner. (A) RT-PCR analysis of cd74 mRNA in the HepG2 cells transfected with scramble (Scr) siRNA or CD74 siRNA. 18s rRNA levels were used as a control. (B) HepG2 cells were pre-treated with compound C (10 μM) for 30 min and were then stimulated with MIF or AICAR for 1 h. Cell lysates were analyzed by Western blotting with an anti-phospho-AMPK(Thr72) antibody. (C) Cells infected with a mock adenovirus or an adenovirus carrying dominant-negative (DN)-AMPK α2 at an MOI of 30 for 18 h were treated with or without MIF. Cell lysates were analyzed by Western blotting. (B, C) Bar graph depicts the mean (+ SE) ratio of intensity of phospho AMPK- to-total AMPK bands. . $^{*}p < 0.05$, $^{**}p < 0.01$ *vs*. control values (one-way ANOVA). Data are expressed as the means ± SD of quadruplicated analyses.
