# Supplementary material for: Involvement of exercise-induced macrophage migration inhibitory factor in the prevention of fatty liver disease
Source: J Endocrinol. 2013 Jul 3;218(3):339–48. doi: 10.1530/JOE-13-0135 (PMC3757527; doi:10.1530/JOE-13-0135)
Supplement: Supplemental Data [file supp_JOE-13-0135_Supplementary_figure_4.pdf]

Supplementary Fig 4.

A.

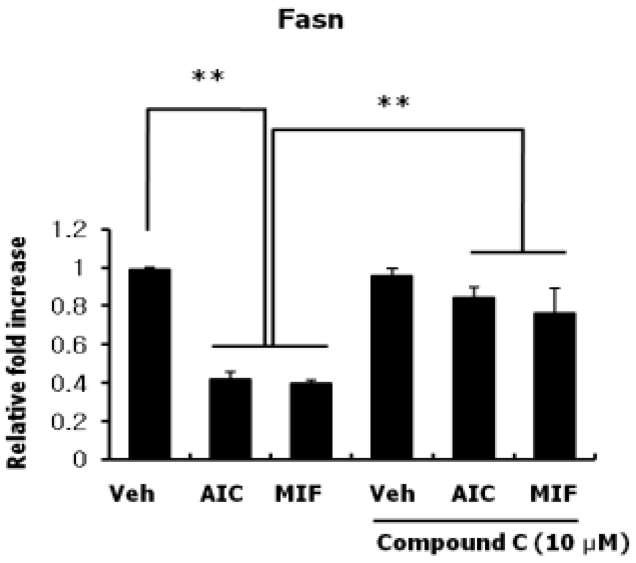

Supplementary Fig. 4. Effect of MIF on the expression of Fasn. (A) HepG2 cells were pre-treated with compound C (10 μM) for 30 min and were then stimulated with MIF or AICAR for 1 h. Cell lysates were analyzed by RT-PCR analysis with Fasn specific primer. 18s rRNA levels were used as a control. **$p < 0.01$ *vs*. control values (one-way ANOVA). Data are expressed as the means ± SD of triplicate analyses.
