# Supplementary material for: Involvement of exercise-induced macrophage migration inhibitory factor in the prevention of fatty liver disease
Source: J Endocrinol. 2013 Jul 3;218(3):339–48. doi: 10.1530/JOE-13-0135 (PMC3757527; doi:10.1530/JOE-13-0135)
Supplement: Supplemental Data [file supp_JOE-13-0135_Supplementary_figure_5.pdf]

Supplementary Fig 5.

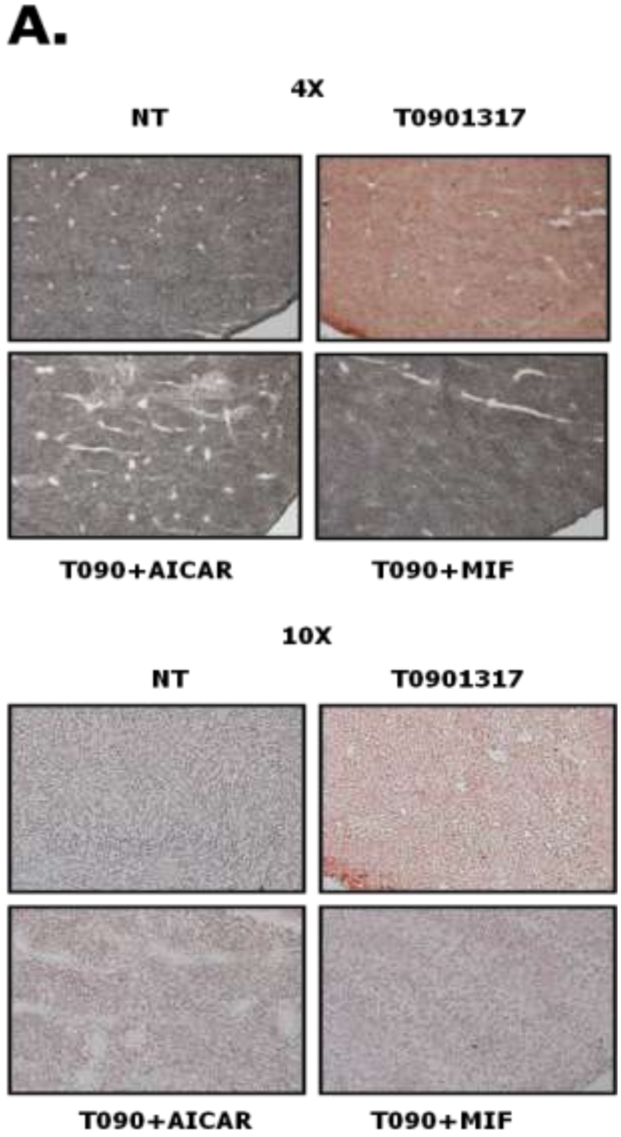

Supplementary Fig. 5. (A) Mice were administrated with Vehicle (saline), AICAR or MIF under conditions of TO901317 (n=4, respectively) stimulation. Intracellular TG contents were measured after 5 day of administration. The mice liver of the each experimental group were prepared for cryosection with optimal cutting temperature (OCT) compound and stained with Oil Red O and hematoxlyin to observe the accumulation of lipids using microscopy (IX71, Olympus, Japan).
